# Antiproliferative and cytostatic effects of the natural product eupatorin on MDA-MB-468 human breast cancer cells due to CYP1-mediated metabolism

**DOI:** 10.1186/bcr2090

**Published:** 2008-05-02

**Authors:** Vasilis Androutsopoulos, Randolph RJ Arroo, John F Hall, Somchaiya Surichan, Gerry A Potter

**Affiliations:** 1 Leicester School of Pharmacy, De Montfort University, The Gateway, Leicester, LE1 9BH, UK

## Abstract

**Introduction:**

The natural product eupatorin has been reported to have antiproliferative activity in tumour cell lines, but the exact mechanism is unclear. The cytochromes P450 CYP1B1, CYP1A1, and CYP1A2 have been shown to participate in the activation of various xenobiotics, compounds derived from the diet as well as chemotherapeutic drugs. CYP1B1 and CYP1A1 have also been proposed as targets for cancer chemotherapy for their differential and selective overexpression in tumour cells. In this study, we aimed to identify a possible mechanism of action for the antiproliferative effect of eupatorin, which can be attributed to CYP1 family-mediated metabolism.

**Methods:**

The study focuses on the antiproliferative action of eupatorin on the human breast carcinoma cell line MDA-MB-468 and on a cell line derived from normal mammary tissue, MCF-10A. The cytotoxicity of the flavone, its effect on the cell cycle of the abovementioned cell lines, and its metabolism by CYP1 family enzymes were examined.

**Results:**

Eupatorin showed a dose-dependent inhibitory effect of cell growth on MDA-MB-468 cells with a submicromolar median inhibition concentration (IC_50_) whereas the IC_50 _of this compound in MCF-10A cells was considerably higher. The antiproliferative effect, as measured by EROD (ethoxyresorufin-*O*-deethylase) assay and Western immunoblotting, was attributed mainly to CYP1A1 expression in MDA-MB-468 cells but not in MCF-10A cells. Moreover, CYP1 family enzymes were shown to metabolise eupatorin *in vitro *to the flavone cirsiliol and two other unidentified metabolites. Metabolism of eupatorin was also detected in MDA-MB-468 cell cultures, whereas metabolism by MCF-10A cells was negligible. Eupatorin was further shown to arrest the cell cycle of the CYP1-expressing cell line MDA-MB-468 in G_2_/M phase, whereas no effect was observed in MCF-10A cells, which do not express CYP1 enzymes. The effect of eupatorin on the MDA-MB-468 cell cycle could be reversed by co-application of the CYP1 inhibitor acacetin.

**Conclusion:**

The flavone eupatorin is selectively activated in breast cancer cells, but not in normal breast cells, due to CYP1 family metabolism. This provides a basis for selectivity which is desired against breast tumour cells. In this sense, eupatorin is shown by this study to be a very promising chemopreventative candidate that should be examined further in an *in vivo *study.

## Introduction

Breast cancer is the most common cancer and the second leading cause of cancer-related death for women in the US [1]. Current chemotherapeutic options for breast cancer include drugs such as tamoxifen and the cytotoxics paclitaxel and docetaxel, all of which show severe side effects [2]. Therefore, there is a focus toward new chemotherapeutic and chemopreventative agents that show limited toxicity to normal tissue and possess a more favourable therapeutic window.

Natural product research has revealed a large variety of phytochemicals that have been proven successful against breast cancer in several epidemiological and *in vitro *studies. In particular, the class of flavonoids has been well established as a group of natural products with versatile anticancer activity. Compounds such as the isoflavone genistein have shown promise in the prevention of breast cancer worldwide [3,4]. Eupatorin is a flavone which is found in various medicinal plants. It is present in *Orthosiphon stamineus*, a plant widely used in southeast Asia for the treatment of various diseases [5], in the leaves of *Lantana montevidensis *(Spreng.) Briq. (Verbenaceae), which is indigenous to tropical regions of the Americas and has been used in folk medicine for the treatment of bronchitis and stomach disorders [6], and in the aerial plants of *Tanacetum vulgare*, a medicinal plant found in South America [7]. Eupatorin has been shown to have anti-inflammatory effects in a mouse ear oedema model [7] as well as antiproliferative activity in human gastric adenocarcinoma (MK-1), human uterus carcinoma (HeLa), and murine melanoma (B16F10) cell lines [6]. In addition, it has demonstrated cytotoxic activity toward highly metastatic murine colon carcinoma (26-L5) cells [5]. However, none of the above studies has proposed a plausible mechanism of action for the antiproliferative or cytotoxic activity of eupatorin.

The cytochromes P450 are enzymes that catalyse the metabolic activation of various xenobiotics. The cytochromes P450 CYP1B1 and CYP1A1 are differentially expressed within the tumour microenvironment compared with the surrounding normal tissue and have been proposed as targets for novel chemotherapeutic agents. The enzymes may catalyse the conversion of prodrugs that are non-toxic to normal cells but are activated to cytotoxic agents in the tumour site [8,9]. CYP1A1 has been shown to be overexpressed in ovarian cancer cells and esophageal tumours, as opposed to normal cells and tissue [10-12], and CYP1B1 protein has been detected in a wide variety of tumours such as breast, brain, colon, and lung, whereas no protein was detected in corresponding normal samples [13]. The natural product resveratrol, which has a structure similar to eupatorin, and the synthetic compounds Phortress and AQ4N are all prodrugs, which are metabolised by CYP1A1 and CYP1B1 to antiproliferative and more active agents [14-16]. The third member of the CYP1 family, CYP1A2, has also been shown to participate in the activation of chemotherapeutic drugs in addition to natural products [17,18]. We therefore undertook a detailed study of the metabolism of the natural flavone eupatorin by CYP1 enzymes *in vitro *and in human breast cancer cells.

A novel mechanism for the antiproliferative action of eupatorin is proposed. The flavone is considered a CYP1A- and CYP1B1-activated prodrug that has cytostatic effects in MDA-MB-468 breast adenocarcinoma cells upon CYP1-mediated metabolism. No cytostatic effects are found in MCF-10A normal breast cells, which do not express CYP1A and CYP1B1. Eupatorin is a natural product that is shown at least in *in vitro *assays to be a potential chemopreventive agent and provides the desired selectivity between cancer and normal cells, a major limitation of standard cytotoxic chemotherapeutic drugs.

## Materials and methods

### Materials

Eupatorin and cirsiliol were purchased from Lancaster (Heysham, Lancashire, UK). Acacetin, nicotinamide adenine dinucleotide phosphate (NADPH), salicylamide, propidium iodide (PI), and phosphate-buffered saline (PBS) were purchased from Sigma-Aldrich (Poole, Dorset, UK), and high-pressure liquid chromatography (HPLC)-grade acetonitrile and methanol were purchased from Fisher Scientific (Loughborough, Leicestershire, UK). Media and cell culture reagents were from Sigma-Aldrich. Western blotting kits, sample buffer, molecular weight marker, nitrocellulose membrane, and filter paper for Western blotting were obtained from Bio-Rad Laboratories (Corston, Bath, UK). Polyvinylidene fluoride (PVDF) membrane was purchased from Sigma-Aldrich. The primary antibodies to detect CYP1A (both CYP1A1 and CYP1A2) and CYP1B1 were obtained from Gentest Corporation (now part of BD Biosciences, San Jose, CA, USA), via BD Biosciences (Cowley, Oxford, UK), and Auvation Limited (Aberdeen, Scotland, UK), respectively. Cell culture media and other ingredients used in lysis buffer in Western blotting, including secondary antibodies, were supplied by Sigma-Aldrich. ECL (enhanced electrochemiluminescence) Plus™ reagent, PVDF membrane, and film used in Western blotting were obtained from Amersham Biosciences (now part of GE Healthcare, Little Chalfont, Buckinghamshire, UK). Microsomes containing human cytochrome P450 (CYP1A1 or CYP1B1) and human NADPH-cytochrome P450 reductase (SUPERSOMES™), prepared from recombinant baculovirus-transformed insect cells, were obtained from BD Biosciences. Control microsomes prepared from insect cells treated with the vector plasmid were also obtained from BD Biosciences.

### Ethical approval

In the UK, by virtue of section 54(7) of the Human Tissue Act, cell lines are not legally regarded as coming from a human body. As such, the experimental use of MDA-MB-468 and MCF-10A cells does not require ethical approval.

### Cell culture

MDA-MB-468 cells were maintained in RPMI-1640 medium without phenol red with 10% foetal calf serum and 2 mM glutamine. MCF-10A cells were maintained in Dulbecco's modified Eagle's medium/Ham's F-12 medium (1:1) with 10% foetal calf serum, 2 mM glutamine, insulin (10 μg/mL), hydrocortisone (500 ng/mL), and epidermal growth factor (20 ng/mL). Cells were passaged every 2 to 3 days using trypsin EDTA (ethylenediaminetetraacetic acid).

### Ethoxyresorufin-*O*-deethylase assay in cells

MDA-MB-468 and MCF-10A cells (5 × 10^4 ^cells per millilitre) were grown in 24-well plates for 48 hours. The ethoxyresorufin-*O*-deethylase (EROD) activity was measured as described previously [19]. The cells were washed once with PBS, and fresh medium containing salicylamide to inhibit conjugating enzymes (1.5 mM) was added to the wells. The plate was left in the incubator at 37°C for 5 minutes, and 7-ethoxyresorufin was added at a final concentration of 5 μM. The reaction was carried out for 1 hour at 37°C with gentle stirring of the plate every 5 minutes. Aliquots (200 μL) were transferred to Eppendorf tubes and the reaction was terminated by the addition of an equal volume of ice-cold methanol, which results in immediate cell lysis. Then, the samples were centrifuged at 3,000 rpm for 10 minutes and the supernatants transferred to a 96-well plate and read using a Spectra Max M5/M5^e ^microplate reader (Molecular Devices Corporation, Sunnyvale, CA, USA) with excitation and emission at 530 and 590 nm, respectively. Standard curves for resorufin formation were also performed.

### Western immunoblotting

MDA-MB-468 and MCF-10A cells were trypsinised and resuspended in 200 μL of lysis solution (Sigma-Aldrich) containing protease inhibitor cocktail (Sigma-Aldrich) and DTT (dithiothreitol) (1 mM). Twenty microlitres of each sample was mixed with SDS-PAGE Laemmli buffer (containing 5% mercaptoethanol) in 1:1 ratio. The samples were heated at 100°C for 4 to 5 minutes and then each sample was loaded to acrylamide gels (10% acrylamide for resolving gel and 5% acrylamide for stacking gel) at the required volume to obtain 15 μg of protein for detection of CYP1A1 or 20 μg of protein for detection of CYP1B1. Wet blotting was used to transfer the protein from the gel to the membrane. For CYP1A detection, nitrocellulose membrane was used, whereas PVDF membrane was used for CYP1B1 detection. The membrane was incubated with 10% milk in 0.05% tris-buffered saline Tween-20 (TBST) at room temperature for 1 hour on the shaker in order to block non-specific binding. Primary antibody was diluted in 5% milk in 0.05% TBST and incubated at 4°C overnight with the membrane. The membrane was then washed six times for 15 minutes each time with 0.05% TBST to rinse out the excess primary antibody and incubated with secondary antibody diluted in 5% milk in 0.05% TBST at room temperature for 1.5 hours. The excess secondary antibody was washed six times with 0.05% TBST as before and the membrane was exposed to ECL Plus™ reagents. Finally, the signal was transferred to a film that was developed in the dark.

### MTT assay

MDA-MB-468 or MCF-10A cells were plated at a density of 10^4 ^cells per millilitre in 96-well flat-bottomed plates. After 24 hours, the medium was carefully aspirated and eupatorin was added in quadruplicate in 200 μL of medium to give a final concentration of not more than 0.1% (vol/vol) dimethylsulfoxide (DMSO). The cells were then allowed to grow for 96 hours at 37°C. The medium was removed and fresh medium with MTT (3-[4,5-dimethylthiazol-2-yl]-2,5-diphenyltetrazolium bromide) (0.4 mg/mL) was added to each well for 3 hours. All medium was aspirated and the formazan product generated by viable cells was solubilised with 150 μL of DMSO. Plates were vortexed and the absorbance at 540 nm determined using a Spectra Max M5/M5^e ^microplate reader. Results were expressed as the percentage of proliferation compared to controls containing 0.1% DMSO. The median inhibition concentration (IC_50_) was calculated using GraphPad Prism software (GraphPad Software, San Diego, CA, USA). The dose range generally was in half-log dilutions (for example, 100, 30, 10, 3, 1, 0.3, 0.1, 0.03, 0.01, 0.003, and 0.001 μM). For inhibition studies, acacetin was added to a final concentration of 1.5 μM in 200 μL of medium along with eupatorin.

### Enzyme assay and high-pressure liquid chromatography analysis

Incubations (100 μL) contained NADPH (0.5 mM), MgCl_2 _(0.5 mM), phosphate buffer (20 mM), and eupatorin (10 μM). The reaction was initiated with the addition of recombinant microsomes expressing CYP1A1 or CYP1B1 (20 pmol/mL of human cytochrome P450) at 37°C and was carried out for 20 minutes. Samples were taken every 5 minutes and each reaction was terminated by the addition of 100 μL of 1% acetic acid in methanol. A centrifugation step at 3,500 *g *for 20 minutes at 4°C ensured that the protein was removed from each sample and the supernatants were analysed by a reversed-phase PerkinElmer 200 HPLC (PerkinElmer, Waltham, MA, USA). For co-elution studies, a 20-minute CYP incubate of eupatorin was prepared as above and spiked with an authentic standard of the putative metabolite. The resulting sample was analysed by HPLC and compared with a control with no metabolite standard.

A Luna 5 μC_18_, 4.6 × 150 mm column (Phenomenex, Macclesfield, Cheshire, UK) was used. The mobile phase consisted of solvent A and solvent B. Solvent A contained 1% acetonitrile and 0.5% acetic acid in water, and solvent B contained 4% acetonitrile and 0.5% acetic acid in methanol. A gradient program was used starting with 60% solvent A and 40% solvent B at time 0 and ending with 10% solvent A and 90% solvent B after 10 minutes. The final conditions were held for 1 minute before returning to initial solvent conditions, and 8 minutes were allowed for column equilibration between each run. Eupatorin and its metabolites were monitored by a Waters 200 UV detector (Waters, Watford, Hertfordshire, UK) at 350 nm. The flow rate was 1 mL/minute and the temperature was 37°C.

### Metabolism in cells

MDA-MB-468 or MCF-10A cells (2 × 10^4 ^cells per millilitre) were plated in 24-well flat-bottomed plates and left to grow for 48 hours. All media were removed and the cells were washed once with PBS. Eupatorin was added in 200 μL of medium at a final concentration of 10 μM and salicylamide at a final concentration of 1.5 mM. The cells were incubated at 37°C for 45 minutes. The medium was removed and eupatorin extracted with 1% acetic acid in methanol. Lysis buffer (200 μL) purchased from Sigma-Aldrich was added to each of the wells and the cells were lysed by continuous pipetting. One percent acetic acid in methanol (200 μL) was added to the cell lysates. Both cell and medium samples were centrifuged for 15 minutes at 13,000 rpm and finally analysed with HPLC. Protein concentration was estimated by the Bradford assay to be 0.5 mg/mL in each sample [20].

### Flow cytometry

MDA-MB-468 or MCF-10A cells were pretreated with eupatorin or cirsiliol or 0.1% DMSO (negative control) for 30 or 48 hours. The medium was aspirated, and the cells were washed with cold PBS, fixed in 70% ethanol, and stored at least 2 hours at -20°C. The cells were then resuspended in PI solution (70 μg/mL in PBS) containing 13 Kunitz units of RNase and incubated at 37°C for 30 minutes. Fluorescence was measured on a fluorescence-activated cell sorter flow cytometer (Beckman Coulter, Oakley Court, Buckinghamshire, UK). The machine was calibrated and the laser aligned using fluorescent beads and a flow-check protocol before each sample was run. All photomultiplier tubes should give a half-coefficient of variance of less than 2.5 in the calibration. All of the parameters required for the DNA analysis protocol, which included voltage, peak gain, integrated gain, and discriminator, were optimised in preliminary experiments. Doublets were separated from single cells in G_2_/M phase by gating. The data obtained from each histogram were then analysed using Multicycle Analysis 2.0 software (Phoenix Flow Systems, San Diego, CA, USA) and compared with manual estimation of the proportion of cells in each phase. Apoptosis was measured by the proportion of cells present in the sub-G_1 _phase.

### Statistical analysis

Results are expressed as mean ± standard deviation for at least three independent experiments, unless indicated otherwise.

## Results

### Eupatorin reversibly inhibits proliferation of MDA-MB-468 cells while having minor effects on MCF-10A cells

**Figure 1:**
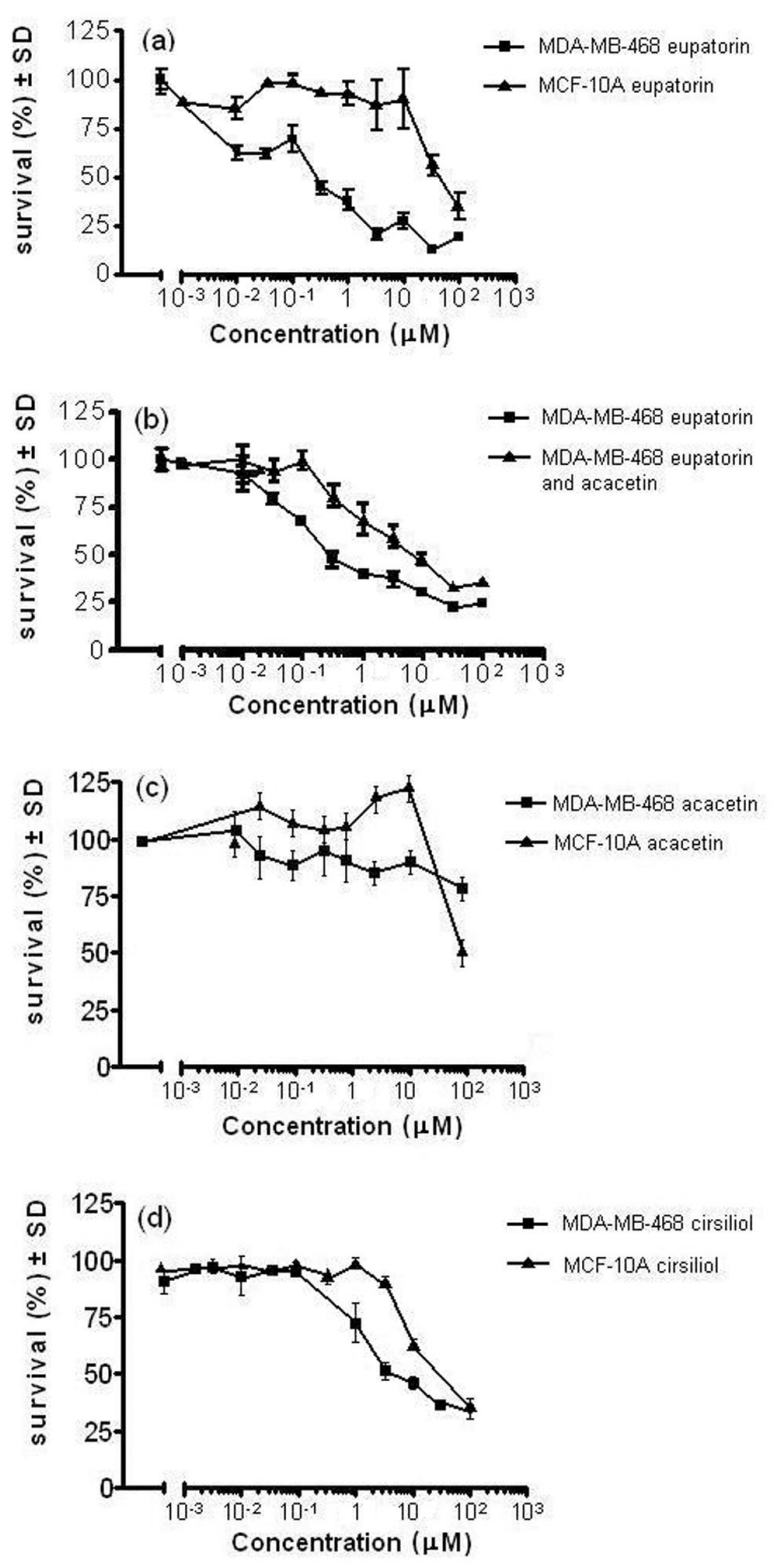
MTT cell proliferation assays. **(a) **Cytotoxicity of eupatorin in MDA-MB-468 cells and MCF-10A cells. **(b) **Decrease of eupatorin cytotoxicity in MDA-MB-468 cells after addition of acacetin. **(c) **Cytotoxicity of acacetin in MDA-MB-468 cells and MCF-10A cells. **(d) **Cytotoxicity of cirsiliol in MDA-MB-468 cells and MCF-10A cells. Cells were plated into 96-well plates and treated with 10^-3 ^to 100 μM eupatorin, acacetin, or cirsiliol (as described in Materials and methods) and allowed to grow for 96 hours. For the inhibition experiment, acacetin was used at a final concentration of 1.5 μM. Error bars represent mean ± standard deviation (SD) for n = 4 determinations. MTT, 3-(4,5-dimethylthiazol-2-yl)-2,5-diphenyltetrazolium bromide.

MDA-MB-468 and MCF-10A cells were treated with eupatorin for 96 hours and cell viability was measured using the MTT assay. MCF-10A is a normal breast cell line that was used as a comparison. Eupatorin had submicromolar toxicity in MDA-MB-468 cells (IC_50 _0.5 μM), whereas it was considerably less active in MCF-10A cells (IC_50 _50 μM) (Figure 1a). To identify whether this effect was due to CYP1 enzyme-mediated metabolism, eupatorin was co-incubated with the CYP1 inhibitor acacetin [21] and MTT assay was carried out as described before. Treatment of MDA-MB-468 cells with both eupatorin and acacetin reversed the cytotoxicity seen when eupatorin was incubated with the cells alone (Figure 1b). When acacetin was added along with eupatorin to MDA-MB-468 cells, a dramatic increase in the IC_50 _was observed (from 0.5 to 15 μM). Acacetin on its own had no effect on cell proliferation (Figure 1c) at the concentration used (1.5 μM). Finally, we examined the antiproliferative effect of the putative eupatorin-CYP1 metabolite cirsiliol and found a profile similar to that of eupatorin. Although cirsiliol was somewhat less potent in MDA-MB-468 cells (IC_50 _2 μM) (Figure 1d), it inhibited MCF-10A proliferation slightly stronger than eupatorin did (IC_50 _15 μM) (Figure 1d).

### CYP1A1 and CYP1B1 proteins are expressed in MDA-MB-468 breast cancer cells

**Figure 2:**
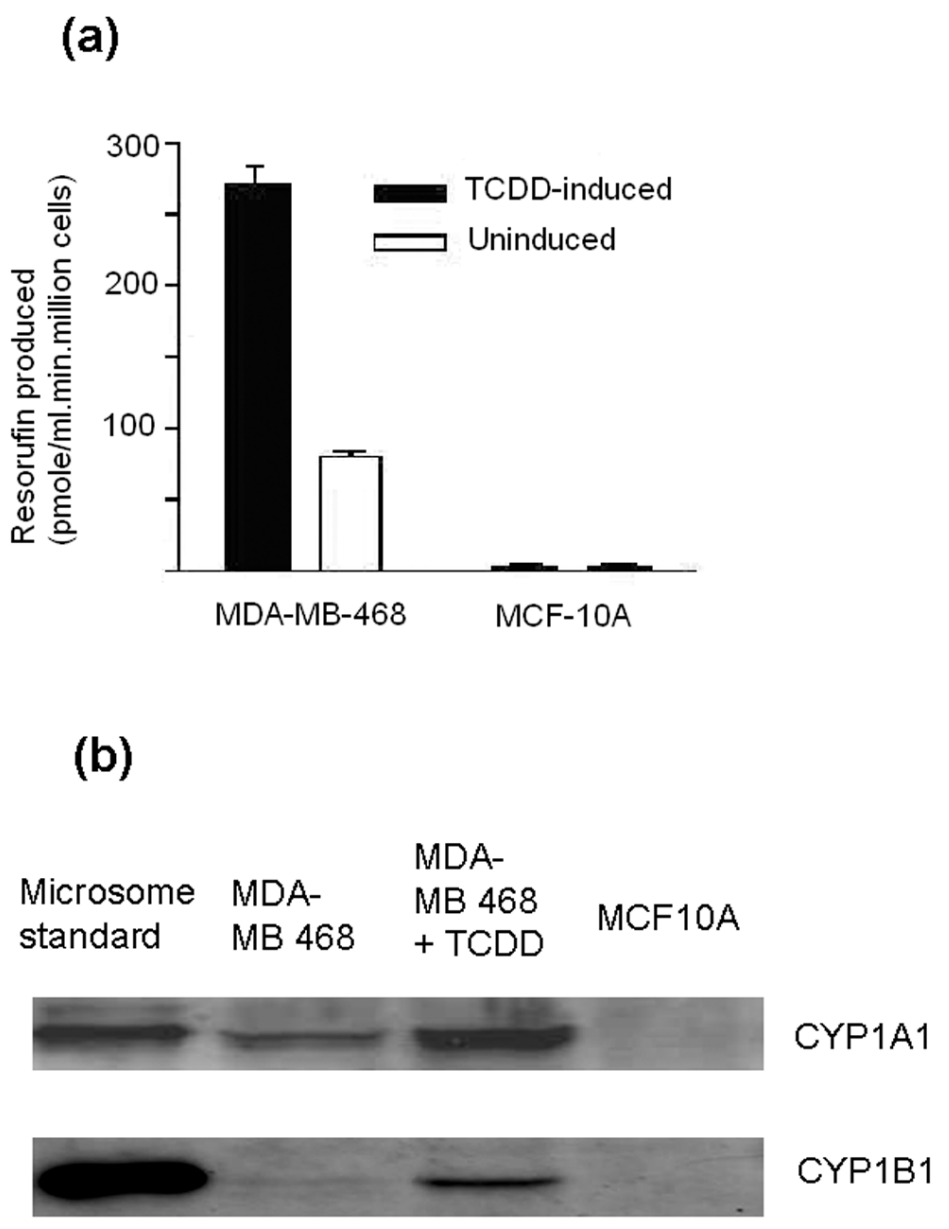
CYP1 enzyme expression in MDA-MB-468 and MCF-10A cells. **(a) **EROD activity of MDA-MB-468 and MCF-10A cells. Cells were seeded at a density of 5 × 10^4 ^cells per millilitre in 24-well plates and left to grow for 48 hours. EROD activity was measured as described in Materials and methods. Error bars represent mean ± standard deviation for n = 4 determinations. **(b) **Selective and inducible CYP1A and CYP1B1 expression in MDA-MB-468 cells. Lysates were probed with anti-CYP1A and anti-CYP1B1 antibodies from Gentest Corporation (now part of BD Biosciences) and Auvation Limited. Lane 1: Recombinant CYP1A1 (top, 0.2 μg) or CYP1B1 (bottom, 0.4 μg) used as positive control. Lane 2: MDA-MB-468 cells. Lane 3: MDA-MB-468 cells treated with 10 nM TCDD for 24 hours. Lane 4: MCF-10A cells. Experiments were performed in duplicate. EROD, ethoxyresorufin-*O*-deethylase; TCDD, 2,3,7,8-tetrachlorodibenzo-*p*-dioxin.

To further support the finding that CYP1 family enzymes are responsible for the bioactivation of eupatorin in MDA-MB-468 and not in MCF-10A cells, the two cell lines were examined for their capacity to express CYP1 family enzymes. CYP1 family enzyme activity was measured in MDA-MB-468 breast cancer and MCF-10A normal breast cells with the EROD assay. The cells were treated with 7-ethoxyresorufin and the amount of resorufin produced was measured by fluorescence. MDA-MB-468 cells clearly expressed active CYP1 family enzymes, whereas very little expression was measured in MCF-10A cells (Figure 2a). Even though the EROD assay is indicative of CYP1A1 activity, it is not specific for any CYP1 enzyme. Thus, Western immunoblotting was employed to provide more information on the CYP1 expression profile of the two cell lines. When the cells were probed with anti-CYP1A1 antibody, MDA-MB-468 cells were shown to express CYP1A1 whereas this protein could not be detected in MCF-10A cells (Figure 2b). A further attempt to examine CYP1B1 protein expression in these two cell lines using an anti-rabbit CYP1B1 antibody revealed a less intense signal of this protein in MDA-MB-468 cells compared with CYP1A1, whereas in MCF-10A cells no CYP1B1 expression was detected (Figure 2b). The expression of CYP1A1 and CYP1B1 proteins was also inducible as treatment with the dioxin TCDD (2,3,7,8-tetrachlorodibenzo-*p*-dioxin) resulted in the upregulation of these two proteins in MDA-MB-468 cells (Figure 2b). The signal for CYP1A1 was stronger than the one observed in the untreated cells and comparable to that of the microsome standard used (Figure 2b). Similarly, CYP1B1 protein level in MDA-MB-468 cells increased after treatment with TCDD, although the signal was considerably less than that of the CYP1B1- and the CYP1A1 microsome standards used. Thus, the breast carcinoma cell line MDA-MB-468 constitutively expresses CYP1A1 and, after induction, increases CYP1A1 levels and expresses low levels of CYP1B1.

### Eupatorin is metabolised to cirsiliol in microsomes expressing human CYP1 family enzymes and in MDA-MB-468 cells

**Figure 3:**
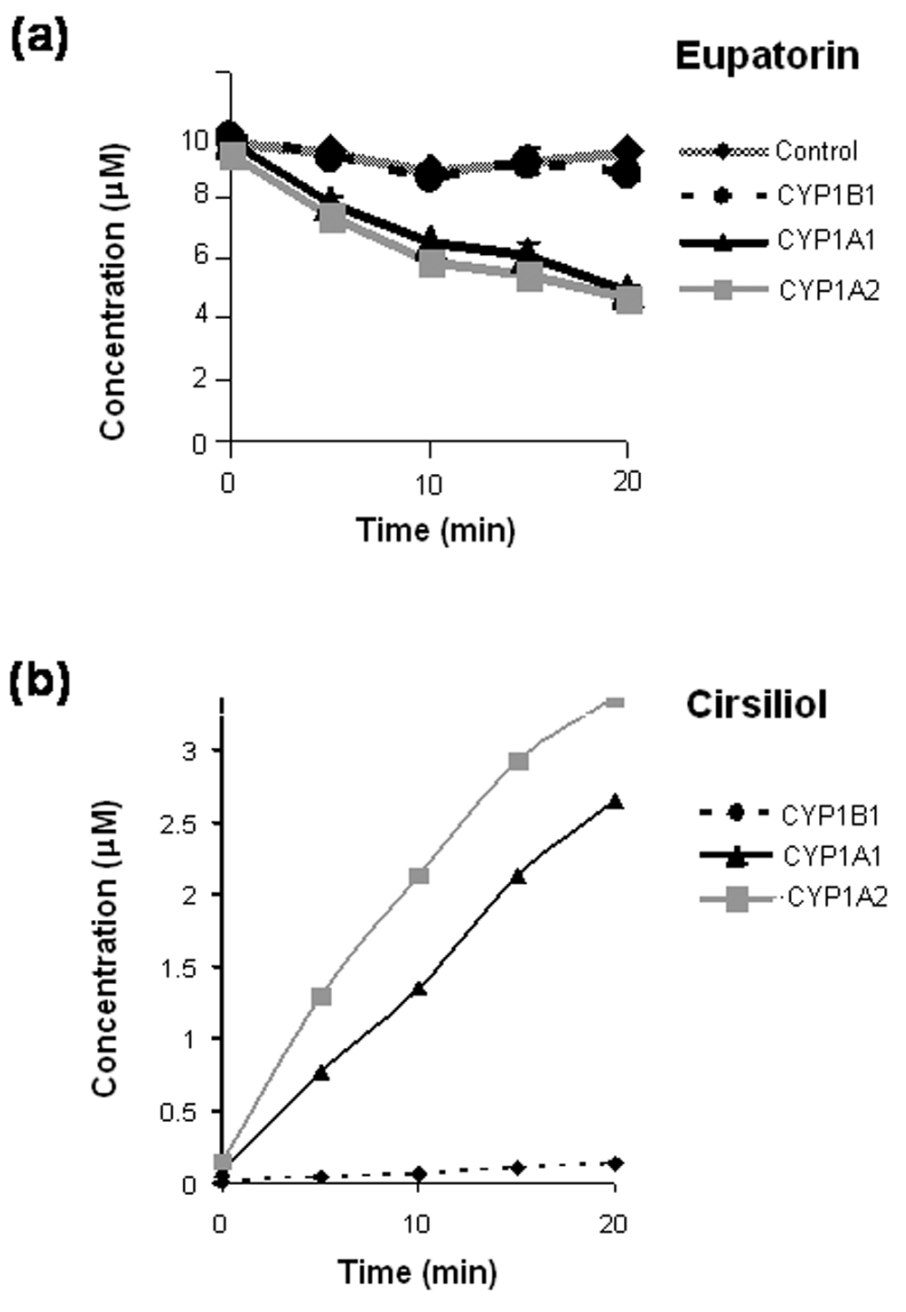
Rate of metabolism of eupatorin (10 μM) by recombinant microsomes expressing CYP1A1, CYP1A2, or CYP1B1. **(a) **Disappearance of eupatorin over time by CYP1 family enzymes. **(b) **Formation of the metabolite cirsiliol over a 20-minute time period. Experiments were performed in duplicate as described in Materials and methods. Error bars represent mean ± minimum or maximum values for n = 2 determinations.

**Figure 4:**
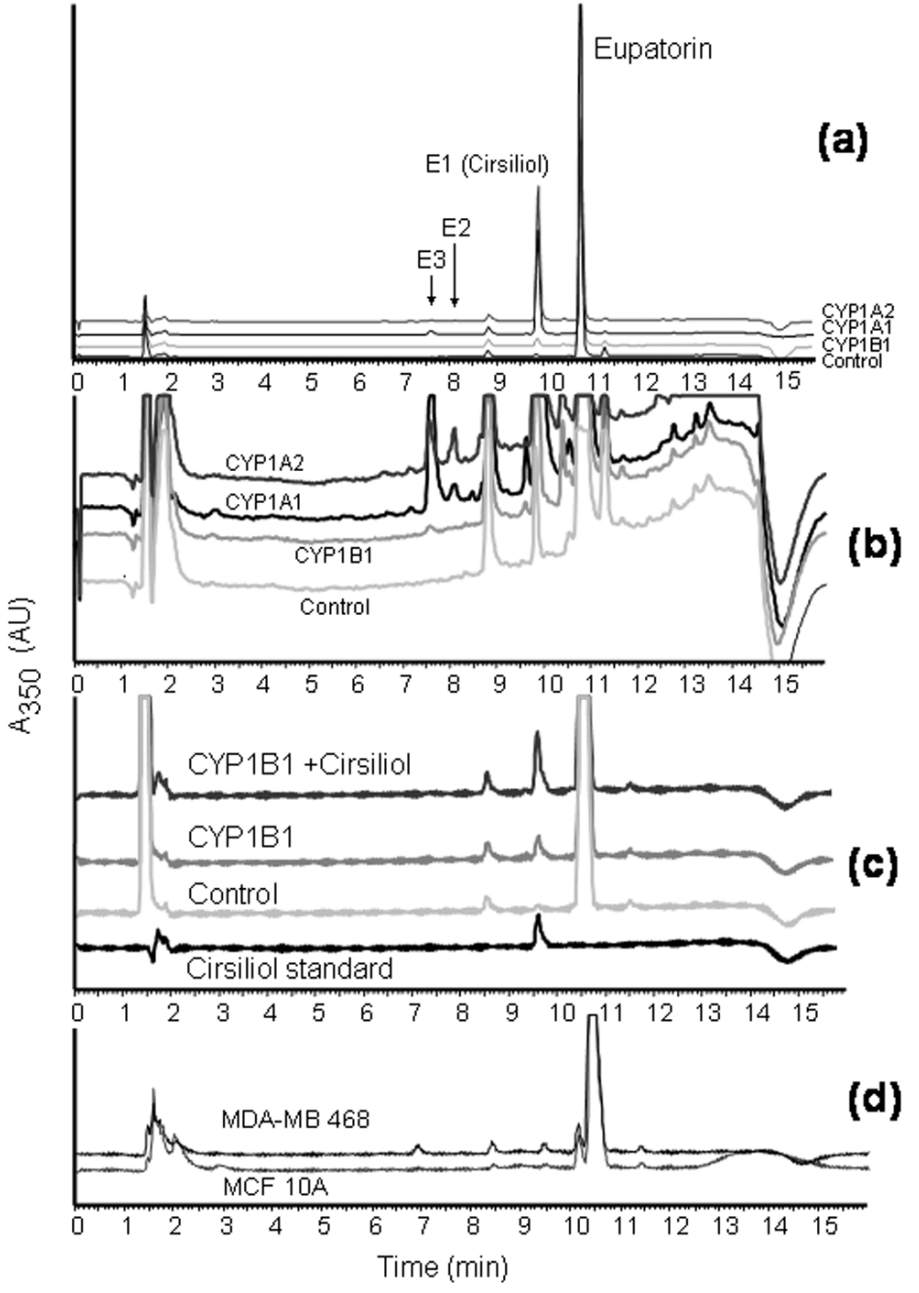
Metabolic profile of eupatorin (10 μM) metabolism by CYP1 family enzymes and identification of cirsiliol as the primary metabolite. **(a) **Typical high-pressure liquid chromatography (HPLC) traces of 20-minute incubation of CYP1 enzymes with eupatorin. **(b) **Expansion of **(a) **showing metabolites E2 and E3. **(c) **Co-elution studies of eupatorin with cirsiliol. A 20-minute CYP1B1 incubate of eupatorin was spiked with cirsiliol (0.2 μM). Reaction mixtures contained eupatorin, NADPH (nicotinamide adenine dinucleotide phosphate), and recombinant microsomes purchased from Gentest Corporation (now part of BD Biosciences). Reactions were terminated by the addition of 1% acetic acid in methanol. **(d) **HPLC trace of metabolism of eupatorin in MDA-MB-468 cells. Samples were analysed by HPLC using a UV detector at 350 nm. Experiments were performed in triplicate. A_350_: absorption of light at wavelength 350 nm. AU: arbitrary units.

Following the first line of evidence that eupatorin is a substrate for CYP1 family enzymes, we investigated the metabolism of this natural product in microsomes expressing human CYP1 family enzymes and in MDA-MB-468 and MCF-10A cells. Microsomes expressing CYP1A1, CYP1A2, or CYP1B1 and NADPH reductase were incubated with eupatorin for 20 minutes with NADPH as a co-factor. Eupatorin was metabolised to a large extent by CYP1A1 and CYP1A2 (Figure 3a). The concentration of the parent compound decreased considerably following incubation with these enzymes, over the 20-minute period, compared with control microsomes that did not contain CYPs. In contrast, CYP1B1 was a weak metaboliser of eupatorin compared with the other two CYP1s (Figure 3a). After incubation with eupatorin, CYP1B1 produced one metabolite, assigned E1, which eluted at approximately 9.6 minutes in the HPLC assay used in this study (Figure 4a). CYP1A1 and CYP1A2 produced three metabolites, assigned E1, E2, and E3, including the one seen in the case of CYP1B1. E2 and E3 were produced in negligible amounts compared with E1 (Figure 4b). All metabolites eluted at retention times shorter than that corresponding to the parent compound. This indicates that they resulted from hydroxylation/demethylation of eupatorin catalysed by the cytochrome P450-reductase system, thereby becoming more polar than eupatorin.

The metabolite eluting at 9.6 minutes was identified as cirsiliol after comparison with a reference standard (Figure 4c) and was the major conversion product of the metabolism of eupatorin by CYP1A1 and CYP1A2 (Figures 3b and 4a). In addition, co-elution studies in which a CYP1B1 incubate of eupatorin was spiked with a small concentration of cirsiliol were performed. This resulted in an increase of the total concentration of cirsiliol in the sample (Figure 4c). The same identification pattern was observed when CYP1A1 was spiked with cirsiliol (data not shown). The metabolites E2 and E3 could not be identified since their authentic standards were not commercially available.

Having shown that eupatorin is converted to cirsiliol by CYP1 family enzymes in microsomal enzyme assays, we conducted cellular assays to find out whether the same or a similar bioconversions occurred. MDA-MB-468 and MCF-10A cells were treated with 10 μM eupatorin for 45 minutes and the samples were analysed with HPLC. Cirsiliol and metabolite E3 could be detected in the MDA-MB-468 cell incubate, but in small quantities compared with the CYP1A1 and CYP1A2 microsome incubates. In contrast, negligible quantities of metabolites were observed in MCF-10A cells (Figure 4d). Thus, the metabolism of eupatorin in MDA-MB-468 cells matched the CYP1A1 enzyme-catalysed metabolism (comparing Figures 4a and 4d).

### Eupatorin causes G_2_/M arrest in MDA-MB-468 cells, as a result of CYP1 family enzyme metabolism, and does not inhibit the cell cycle of MCF-10A cells

**Figure 5:**
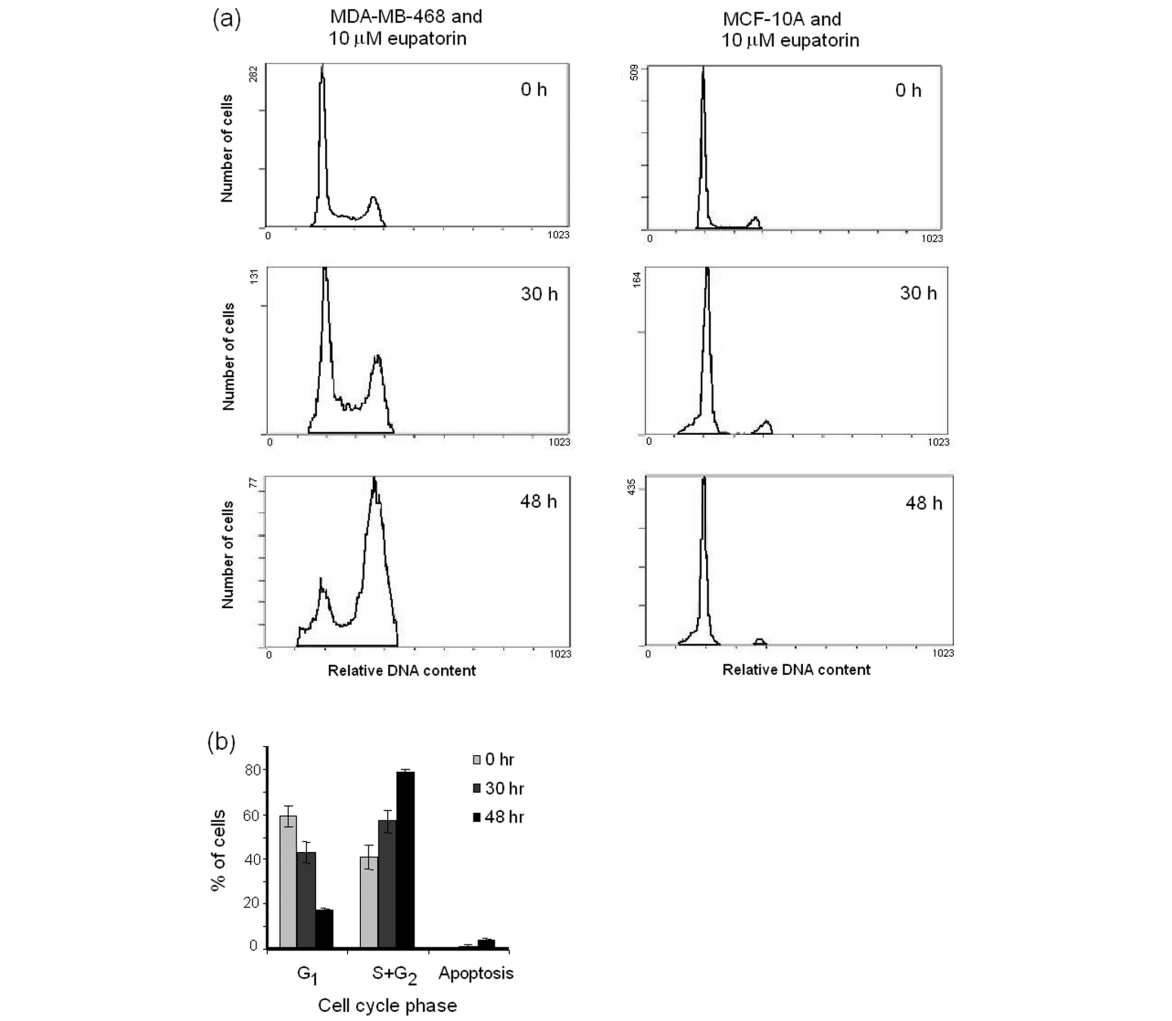
Eupatorin causes G_2_/M arrest in MDA-MB-468 cells, whereas this effect is not observed in MCF-10A cells. **(a) **Flow cytometric DNA analysis of MDA-MB-468 and MCF-10A cells treated with 10 μM eupatorin. **(b) **Percentage of MDA-MB-468 cells in G_1_, S+G_2_/M, and sub-G_1 _phases of the cell cycle. Cells were plated in 24-well plates and left to grow for 48 hours. Eupatorin was incubated with the cells for 30 and 48 hours, and 0.1% dimethylsulfoxide was used as a control. The cells were stained with propidium iodide and analysed using a Beckman Coulter flow cytometer as described in Materials and methods. Error bars represent mean ± standard deviation for n = 3 determinations.

**Figure 6:**
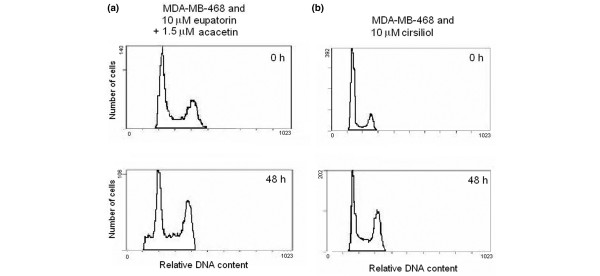
G_2_/M arrest of MDA-MB-468 caused by eupatorin is due in part to metabolism to cirsiliol and can be reversed by acacetin. **(a) **Cell cycle analysis of MDA-MB-468 cells co-treated with 10 μM eupatorin and 1.5 μM acacetin for 48 hours. Histograms are one trace of three independent experiments. **(b) **Cell cycle profile of MDA-MB-468 cells treated with 10 μM cirsiliol for 48 hours. The experiment was performed in duplicate.

Flow cytometry was used to study the effect of eupatorin on the cell cycle of MDA-MB-468 and MCF-10A cells. The purpose of the experiments was to provide more insight into the mechanism of action of the antiproliferative effect of eupatorin and how this could be related to the CYP1 enzyme-mediated metabolism. In addition, we wanted to identify which of the metabolites is mainly responsible for the potency of eupatorin observed in MDA-MB-468 cells. Cells were treated with 10 μM eupatorin for 30 hours, which resulted in a block in the G_2_/M phase of the cell cycle compared with control cells treated with 0.1% DMSO (Figure 5a). This effect was more profound in the 48-hour period, during which approximately 70% of the cells accumulated in the G_2_/M phase (Figure 5b). Furthermore, eupatorin caused apoptosis, which was also enhanced from 30 to 48 hours, as is shown by the sub-G_1 _peak (Figure 5a,b). However, this effect was negligible compared with the G_2_/M block. To prove that the cell cycle arrest was due to metabolism of eupatorin by CYP1 enzymes, we co-treated MDA-MB-468 cells with 10 μM of the compound and 1.5 μM acacetin for 48 hours. Acacetin (1.5 μM) reversed the population of MDA-MB-468 cells arrested in G_2_/M from 70% to 40% but failed to completely return the cell cycle to the initial form observed in the control sample (Figure 6a). Acacetin (3 μM) showed a similar effect (data not shown). Acacetin also failed to correct the phenomenon of apoptosis noticed after treatment of MDA-MB-468 cells with eupatorin. In MCF-10A cells, eupatorin did not cause cell cycle arrest (Figure 5a). However, a small fraction of the cells had undergone apoptosis after 30 hours (5% of cells) and 48 hours (6% of cells) (Figure 5a). Thus, apoptosis was an intrinsic effect of the compound and did not result from CYP1 family enzyme metabolism.

Eupatorin caused G_2_/M arrest in MDA-MB-468 cells, and the effect could be reversed by co-incubation with the CYP1 inhibitor acacetin. Thus, the G_2_/M arrest must have been due to CYP1-mediated metabolism, but which of the metabolites was responsible for this effect remained unknown. Since cirsiliol was the only metabolite that had been identified in our analysis, we treated MDA-MB-468 cells with this compound for 30 and 48 hours and then examined the cell cycle by using flow cytometry. Cirsiliol also caused G_2_/M arrest of MDA-MB-468 cells after 48 hours (Figure 6b). However, the effect was not as dramatic as that observed with eupatorin. The percentage of cells in G_2_/M increased from 24% in control to 39% in cirsiliol-treated MDA-MB-468 cells.

## Discussion

The flavonoids are widely recognised as a class of natural products with cancer-protective properties. In the present study, it is shown that the flavone eupatorin strongly inhibits proliferation of MDA-MB-468 cells at submicromolar concentrations as a result of CYP1 family enzyme metabolism, which suggests that it may be a candidate chemopreventative compound toward breast cancer cells that express CYP1 enzymes. Importantly, we found that eupatorin was non-toxic in human normal breast MCF-10A cells at low concentrations, and it was only at the highest concentrations (>10 μM) that cell viability was affected.

The differential expression of cytochrome P450s such as CYP1B1 protein in breast tumour samples, as opposed to normal tissue, has been proposed as a target for cancer chemotherapy. However, there are often difficulties associated with relating protein expression to functional activity of CYP1A1 and CYP1B1 [16], and to our knowledge there is no direct conventional assay that can differentiate between the activities of these two enzymes. According to our data, MDA-MB-468 cells expressed both enzymes at the protein level. CYP1B1 protein was detected at very low levels, whereas CYP1A1 protein expression was much stronger. Their expression was also upregulated following treatment with the CYP1 inducer TCDD. MDA-MB-468 cells have previously been used to examine the effects of the antitumour agent DF203 on the regulation of CYP1A1 and CYP1B1 proteins [22]. In that study, MDA-MB-468 cells were said to constitutively express CYP1B1, but not CYP1A1, to the protein level by using polyclonal rabbit anti-human CYP1B1 and polyclonal antiserum for human CYP1A1/1A2, respectively [22]. In the present study, we could not detect any cross-reaction of the CYP1A1 antibodies we used with CYP1B1-containing microsomes (data not shown). It is possible that the CYP1A1 antibody used in the study of Chua and colleagues [22] was not specific enough to detect the desired protein. Importantly, MCF-10A cells expressed neither CYP1A nor CYP1B1 at the protein level.

MDA-MB-468 cells constitutively express CYP1A1 enzymes in their active form. Some authors have reported that the estrogen receptor-negative breast cancer cell line MDA-MB-468 expresses CYP1A1 active protein only after treatment with the CYP1 inducer TCDD [23]. However, low levels of CYP1B1 and CYP1A1 activity have been reported for similar breast cancer cell lines such as MDA-MB-436, MDA-MB-231, MDA-MB-157, and T47 in the absence of TCDD, whereas in its presence the activity was largely augmented [24]. According to our results, MDA-MB-468 cells expressed active CYP1A1 in the absence of an inducer. The activity was considerably high compared with the normal breast cell line MCF-10A but was about three orders of magnitude less than that of MCF-7 cells induced with 10 nM TCDD for 24 hours (data not shown). Hence, MDA-MB-468 can be used as a model cell line to test the possible bioactivation of xenobiotics by CYP1A1. It should be noted, however, that after induction CYP1B1 activity may also play a role in cell metabolism.

CYP1A1 has been involved in the activation of two main chemotherapeutic agents in breast cancer cell lines. In the human breast adinocarcinoma cell line MCF-7, the anticancer compound DF203 induces CYP1A1 expression. In turn, the enzyme activates its inducer to a more cytotoxic product, which results in a remarkable IC_50 _of 10 nM under these conditions [22]. However, when the CYP1 inhibitor α-napthoflavone [25] is co-incubated with the drug, the IC_50 _is greater than 100 μM in MCF-7 cells. Aminoflavone is an antitumour drug that acts in a similar way [26]. Again, CYP1A1 is responsible for its activation. This agent has astonishing IC_50 _values of 0.1 nM in MCF-7 cells and 600 nM in AH^R100 ^cells, an AhR-deficient variant of MCF-7 cells, where the enzyme CYP1A1 is not induced [26]. Unlike the abovementioned compounds, which show activation factors of greater than 1,000, eupatorin had only a 30-fold difference in IC_50 _when it was co-incubated with the CYP1 inhibitor acacetin. This demonstrates that it would be more effective as a chemopreventative rather than as a chemotherapeutic drug. Regardless of the difference in activity, our study reinforces the pivotal role of CYP1 enzymes and mainly CYP1A1 in breast cancer prevention and therapy through the activation of xenobiotics.

A recent study examined the antitumour activity of a new series of fluoro-, methoxyl-, and amino-substituted isoflavones in human breast MDA-MB-468 and MCF-7 cells as well as two colon cancer cell lines [27]. The authors reported that MDA-MB-468 cells were the most sensitive overall, and significant potentiation of growth inhibitory activity (GI_50 _< 1 μM) was observed for some of these compounds when MDA-MB-468 cells were co-incubated with the CYP1A1 inducer TBDD (2,3,7,8-tetrabromo dibenzo-p-dioxin), which suggests a possible CYP1A1 bioactivation of the isoflavone derivatives [27].

Eupatorin has been shown to inhibit proliferation of human gastric adenocarcinoma (MK-1), human uterus carcinoma (HeLa), and murine melanoma (B16F10) cell lines [6]. However, in the literature, there is no additional information regarding the antiproliferative mechanism of action of this compound. In the present study, the antiproliferative activity of eupatorin found in MDA-MB-468 cells is attributed to a large extent to its metabolism by the CYP1 family enzymes. First, we observed that the CYP1 enzyme inhibitor acacetin considerably increased the IC_50 _of eupatorin in MDA-MB-468 cells. Second, all CYP1 enzymes metabolised eupatorin to cirsiliol, and CYP1A1 and CYP1A2 metabolised it to another two unidentified metabolites, as shown in the microsomal enzyme assay system. Moreover, in MDA-MB-468 cells, eupatorin was metabolised to cirsiliol and the metabolite E3, whereas no metabolism could be detected in MCF-10A cells.

**Figure 7:**
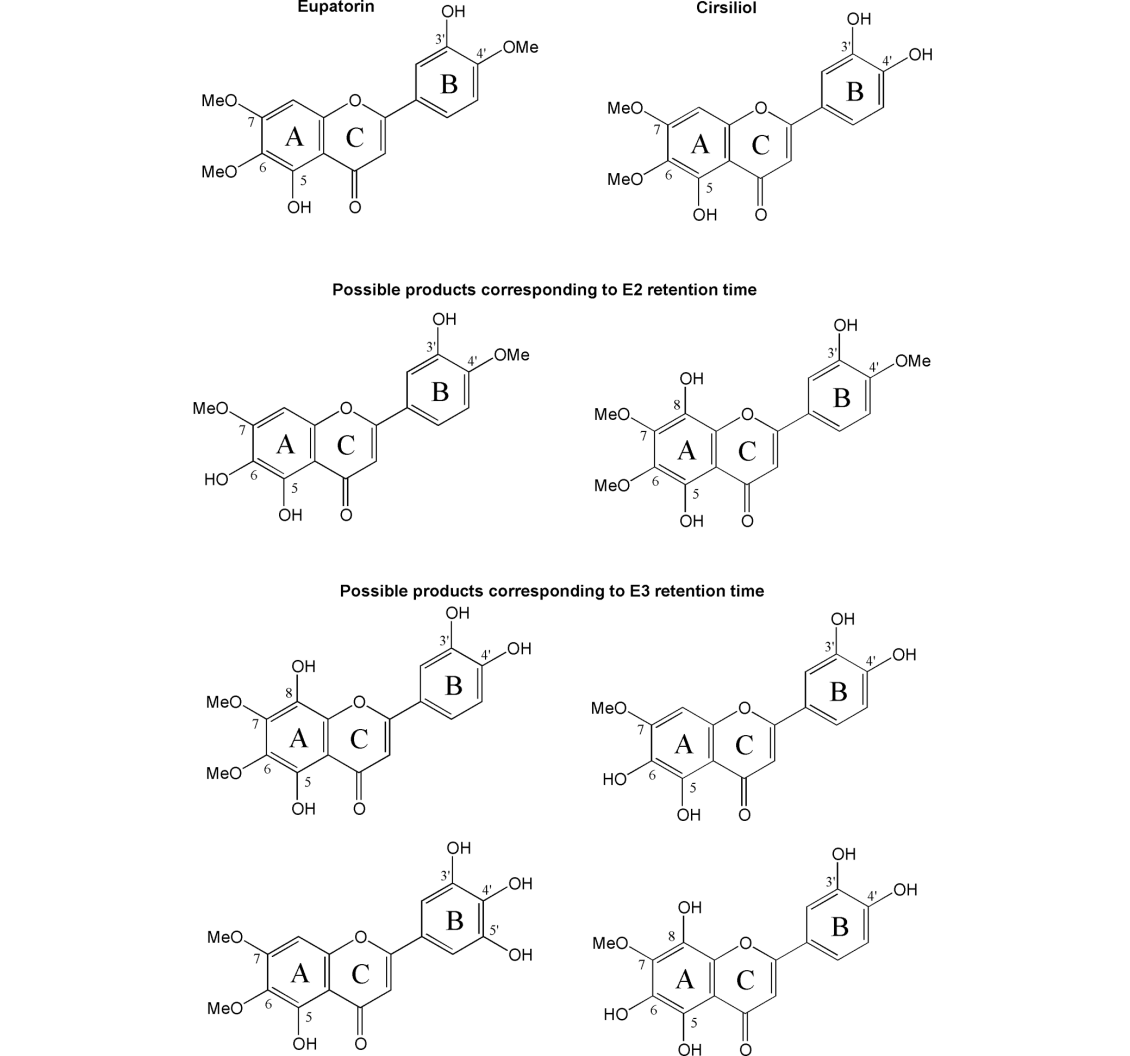
Chemical structures of eupatorin and cirsiliol and possible structures of the metabolites E2 and E3.

Cirsiliol, or 3',4'-dihydroxy-5,6,7-trimethoxy flavone (Figure 7), results from 4'-demethylation of eupatorin. The IC_50 _values for cirsiliol in MDA-MB-468 and MCF-10A cells show a 7-fold difference (Figure 1d), which indicates that CYP1-mediated metabolism further activates this compound. Nevertheless, this initial eupatorin metabolite is at least partially responsible for its antiproliferative activity as it showed lower IC_50 _values in MCF-10A cells than eupatorin did. In addition, the metabolites E2 and E3 contribute to the ultimate submicromolar toxicity of eupatorin in MDA-MB-468 cells, which was not observed for cirsiliol. The unidentified metabolites E2 and E3 may be produced by hydroxylation reactions at positions 5' of the B ring and 8' of the A ring or by demethylation reactions at positions 6' and 7' of the A ring (Figure 7).

We have not been able to find any reports on CYP1-mediated or other cytochrome P450-mediated metabolism of eupatorin. However, cirsiliol was found to be 3-fold and 5-fold more active than eupatorin in MK-1 and B16F10 cells, respectively [6]. Thus, substitution of the 4'-OCH_3 _to the 4'-OH group in the B ring of eupatorin dramatically alters its antiproliferative activity. In addition, there is increasing evidence that flavones structurally similar to eupatorin are substrates for CYP1 enzymes. Previous studies have underlined the importance of 4'-OCH_3 _demethylation by CYP1A1, CYP1A2, and CYP1B1 as the main metabolic route of flavones and flavonols containing one methoxy group [21,28,29]. This type of conversion has been deemed as a bioactivation because the metabolite was expected to have greater biological activity than the parent compound. A recent study has demonstrated that hydroxylation of the isoflavone genistein in MCF-7 cells as a result of CYP1A1- and CYP1B1-mediated metabolism can enhance its antiproliferative properties [18]. Our results agree with the above findings.

In combination with HPLC analysis of CYP1 enzyme metabolism and cytotoxic assays of eupatorin, flow cytometry demonstrated that eupatorin arrests MDA-MB-468 cells in the G_2_/M phase of the cell cycle as a result of cytochrome P450 metabolism. Cirsiliol is a primary product but is not entirely responsible for this effect since it produced only a moderate inhibition of the cell cycle at G_2_/M. Thus, the profound effect of eupatorin in MDA-MB-468 cells has to be attributed to all of its metabolites. Some flavonoids with a structure similar to eupatorin are known to have antimitotic activity. For example, 5,3'-dihydroxy-3,6,7,8,4'-pentamethoxy flavone inhibits tubulin polymerisation [30]. Casticin, or 5 hydroxy 4', 3,6,7-tetramethoxy flavone, has also been shown to disrupt mitotic spindle morphology of human KB carcinoma cells [31]. A recent study has underlined that the 3-hydroxy-4-methoxy groups of the B ring in synthetic chalcones (which are equivalent to the 3' and 4' positions on the B ring in flavonoids) are crucial in enhancing the binding to the colchicine site in tubulin [32]. Metabolism of eupatorin by CYP1A1 and CYP1B1 might create 3',4'-dihydroxy or 3'-hydroxy-4'-methoxy groups in positions that will strongly bind to the colchine-binding site in tubulin and inhibit mitotic spindle formation. Further studies are required to confirm this hypothesis.

In conclusion, the results presented in this study provide more evidence about the potential of the cytochromes P450 CYP1A1 and CYP1B1 as targets in cancer therapy and prevention. CYP1-catalysed hydroxylation of the natural product eupatorin in breast cancer cells results in bioactivation to cytostatic, and possibly antimitotic, agents. Importantly, this process has no effect on normal breast cells, which do not express CYP1 enzymes. Eupatorin is shown by this to be a promising chemopreventative candidate because of its selective bioactivation in tumour cells which and should be further evaluated by *in vitro *mechanistic assays and *in vivo *studies.

## Conclusion

In summary, this study reports the first preliminary evidence on the antiproliferative mechanism of action of the natural flavone eupatorin on human breast carcinoma cells. The compound is shown to be selective toward the tumour cells, and its cell growth-inhibiting activity is attributed to CYP1 family-mediated metabolism.

## Abbreviations

DMSO = dimethylsulfoxide; ECL = enhanced electrochemiluminescence; EROD = ethoxyresorufin-*O*-deethylase; HPLC = high-pressure liquid chromatography; IC_50 _= median inhibition concentration; MTT = 3-(4,5-dimethylthiazol-2-yl)-2,5-diphenyltetrazolium bromide; NADPH = nicotinamide adenine dinucleotide phosphate; PBS = phosphate-buffered saline; PI = propidium iodide; PVDF = polyvinylidene fluoride; TBST = tris-buffered saline Tween-20; TCDD = 2,3,7,8-tetrachlorodibenzo-*p*-dioxin.

## Competing interests

The authors declare that they have no competing interests.

## Authors' contributions

VA participated in the preparation of the manuscript, the experimental design, and the acquisition, analysis, and interpretation of data. RA was involved in drafting the manuscript and revising it critically for important intellectual content. JH made a substantial contribution to the analysis and interpretation of flow cytometry data. SS participated in the study design and the acquisition, analysis, and interpretation of the MTT data and immunoassays. GP made substantial contributions to the conception and design of the experiments. All authors read and approved the final manuscript.
